# Quality of Acute Psychedelic Experience Predicts Therapeutic Efficacy of Psilocybin for Treatment-Resistant Depression

**DOI:** 10.3389/fphar.2017.00974

**Published:** 2018-01-17

**Authors:** Leor Roseman, David J. Nutt, Robin L. Carhart-Harris

**Affiliations:** Psychedelic Research Group, Department of Medicine, Imperial College London, London, United Kingdom

**Keywords:** psychedelics, psilocybin, depression, therapy, mystical experience, peak experience, serotonin

## Abstract

**Introduction:** It is a basic principle of the “psychedelic” treatment model that the quality of the acute experience mediates long-term improvements in mental health. In the present paper we sought to test this using data from a clinical trial assessing psilocybin for treatment-resistant depression (TRD). In line with previous reports, we hypothesized that the occurrence and magnitude of Oceanic Boundlessness (OBN) (sharing features with mystical-type experience) and Dread of Ego Dissolution (DED) (similar to anxiety) would predict long-term positive outcomes, whereas sensory perceptual effects would have negligible predictive value.

**Materials and Methods:** Twenty patients with treatment resistant depression underwent treatment with psilocybin (two separate sessions: 10 and 25 mg psilocybin). The Altered States of Consciousness (ASC) questionnaire was used to assess the quality of experiences in the 25 mg psilocybin session. From the ASC, the dimensions OBN and DED were used to measure the mystical-type and challenging experiences, respectively. The Self-Reported Quick Inventory of Depressive Symptoms (QIDS-SR) at 5 weeks served as the endpoint clinical outcome measure, as in later time points some of the subjects had gone on to receive new treatments, thus confounding inferences. In a repeated measure ANOVA, Time was the within-subject factor (independent variable), with QIDS-SR as the within-subject dependent variable in baseline, 1-day, 1-week, 5-weeks. OBN and DED were independent variables. OBN-by-Time and DED-by-Time interactions were the primary outcomes of interest.

**Results:** For the interaction of OBN and DED with Time (QIDS-SR as dependent variable), the main effect and the effects at each time point compared to baseline were all significant (*p* = 0.002 and *p* = 0.003, respectively, for main effects), confirming our main hypothesis. Furthermore, Pearson's correlation of OBN with QIDS-SR (5 weeks) was specific compared to perceptual dimensions of the ASC (*p* < 0.05).

**Discussion:** This report further bolsters the view that the quality of the acute psychedelic experience is a key mediator of long-term changes in mental health. Future therapeutic work with psychedelics should recognize the essential importance of *quality of experience* in determining treatment efficacy and consider ways of enhancing mystical-type experiences and reducing anxiety.

**Trial Registration:** ISRCTN, number ISRCTN14426797, http://www.isrctn.com/ISRCTN14426797

## Introduction

Psychedelic therapy may be more appropriately thought of as a distinct form of (drug-assisted) psychotherapy than as a pure pharmacotherapy. Psychedelic therapy involves a small number of high-dose psychedelic dosing sessions that are intended to facilitate a profound, potentially transformative psychological experience (Dyck, 2006; Majić et al., 2015). Psychedelic dosing sessions do not take place in isolation but rather are flanked by psychological preparation and integration. Preparation is intended to facilitate trust and rapport and a mind-set tuned toward emotional openness and “letting go” of psychological resistance (Richards, 2015; Russ and Elliott, 2017). Dosing sessions themselves typically take place in a welcoming environment, with dim lighting, eye-shades, calming and emotionally-directing music, with empathic support provided by trained therapists. The integration sessions subsequent to the dosing session(s) involve the same therapists (usually two) listening to the patient's narrative of their experience, which may include e.g., details of specific emotional insights.

A guiding principle of psychedelic psychotherapy is that the occurrence of a profound, potentially transformative psychological experience is critical to the treatment's efficacy. Evidence has shown that high-dose psychedelic sessions can reliably produce profound psychological experiences rated among the most “meaningful” of a person's life (Griffiths et al., 2006). A number of research teams have referred to these profound experiences and have applied relevant rating scales that have evolved out of studies of spontaneous and drug-induced “mystical,” “spiritual,” “peak” or “religious” experiences (Maslow, 1959; Stace, 1960; Pahnke and Richards, 1966; Maclean et al., 2012). Regardless of the terms chosen to define them, evidence suggests that profound psychological experiences can be predictive of subsequent psychological health, whether induced by psychedelics (O'Reilly and Funk, 1964; Klavetter and Mogar, 1967; Pahnke et al., 1970; Kurland et al., 1972; Richards et al., 1977; Maclean et al., 2011; Garcia-Romeu et al., 2014; Bogenschutz et al., 2015; Griffiths et al., 2016; Johnson et al., 2016; Ross et al., 2016), or other means (James, 1902; Maslow, 1959; Noyes Jr, 1980; Ludwig, 1985; Csikszentmihalyi and Csikszentmihalyi, 1992; Snell and Simmonds, 2015). Furthermore, some recent ketamine for depression studies have also found an association between the quality of acute experience (Sos et al., 2013; Luckenbaugh et al., 2014)—including the occurrence of mystical-type experiences (Dakwar et al., 2014)—subsequent positive clinical outcomes. Given the growing evidence favoring the therapeutic value of psychedelics (dos Santos et al., 2016; Rucker et al., 2016; Carhart-Harris and Goodwin, 2017), it is timely that we better understand their therapeutic mechanisms.

The so-called “mystical” experience has been a classic problem area for mainstream psychology—if not science more generally. The term “mystical” is particularly problematic, as it suggests associations with the supernatural that may be obstructive or even antithetical to scientific method and progress (Carhart-Harris and Goodwin, 2017). It is important to note that by using the term the mystical-type experience, we are referring only to the phenomenology of the experience and are keen not to endorse any associations between it and supernatural or metaphysical ideas. Readers interested in the phenomenology of mystical-type/peak experiences may wish to explore these classic texts (James, 1902; Stace, 1960; Maslow, 1964; Pahnke and Richards, 1966; Csikszentmihalyi and Csikszentmihalyi, 1992; Hood Jr et al., 2009; Richards, 2015).

In the late 1960s, William Richards and Walter Pahnke (former pupils of Abraham Maslow and Timothy Leary respectively) developed a measure of “peak” or “mystical-type” experience that was much inspired by the work of Stace (1960). Studying reports of “mystical-type” experiences occurring in a variety of different world religions, Stace identified a number of common or “universal” components that are largely independent of religious or cultural context (Stace, 1960). Based on this landmark work, Richards and Pahnke developed the “mystical experience questionnaire” (MEQ) designed to enquire whether related components featured in the psychedelic drug experience. The scale measured six components of experience: (1) sense of unity or oneness, (2) transcendence of time and space, (3) deeply felt positive mood, (4) sense of awesomeness, reverence and wonder, (5) meaningfulness of psychological or philosophical insight, (6) ineffability and paradoxicality (Pahnke and Richards, 1966; Pahnke et al., 1970). A similar questionnaire which is based on Stace (1960) is the “M scale” (Hood Jr, 1975). Both the MEQ and M scale have been found to be predictive of long-term positive therapeutic outcomes in trials of psilocybin for cancer-related distress (Griffiths et al., 2016; Ross et al., 2016), tobacco smoking (Garcia-Romeu et al., 2014; Johnson et al., 2016) and alcohol dependence (Bogenschutz et al., 2015).

Perhaps the most widely used subjective measure of altered states of consciousness, and particularly the psychedelic state, is the altered states of consciousness questionnaire (ASC) (Dittrich, 1998). We chose this scale over the MEQ as it measures a broader range of subjective phenomena, not just the “mystical-type experience.” Crucially, this enabled us to test the *specificity* of the relationship between mystical-type experiences (vs. e.g., perceptual changes) and subsequent therapeutic outcomes. One of the principal ASC factors is named “oceanic boundlessness” (OBN)—a term that has its origins in a conversation between Sigmund Freud and the French intellectual and “mystic” Romain Rolland (Freud, 1920) and makes reference to an “oceanic feeling” of boundlessness (Freud, 1929). Sharing a common intellectual background in Stace (1960) (Majić et al., 2015), items belonging to the OBN are closely related to those found in the MEQ. Previous factor analyses have parcellated the ASC into either 5 (Dittrich, 1998) or 11 dimensions (Studerus et al., 2010). As one of the original 5 ASC factors, OBN is explicitly linked to Stace's “mystical experience”, (Studerus et al., 2010) and 4 of the 11 revised ASC factors also relate to OBN. Explicitly, the 4 OBN sub-factors are named “insightfulness,” “blissful state,” “experience of unity” and “spiritual experience” (Studerus et al., 2010).

We recently completed an open-label clinical trial assessing the feasibility of treating 20 patients with treatment-resistant depression (TRD) with psilocybin (Carhart-Harris et al., 2017). Results were encouraging: 47% of patients showed a clinically significant response 5 weeks post treatment (≥50% reduction in depressive symptoms). The present study sought to extend on our previous reports on this trial, by specifically focusing on whether the quality of the acute psychedelic experience was predictive of longer-term clinical outcomes. Specifically, we asked whether psilocybin-induced OBN and Dread of Ego Dissolution (DED) (related to acute anxiety) were predictive of decreases in depression at a key endpoint, whether the relationship between OBN and decreased depression was significantly stronger than between psilocybin's more generic sensory perceptual effects and depression changes.

## Materials and methods

This trial received a favorable opinion from the National Research Ethics Service London—West London, was sponsored and approved by Imperial College London's Joint Research and Compliance Office (JRCO), and was adopted by the National Institute for Health Research Clinical Research Network. The National Institute for Health Research/Wellcome Trust Imperial Clinical Research Facility gave site-specific approval for the study. The study was reviewed and approved by the Medicines and Healthcare products Regulatory Agency (MHRA) and a Home Office Schedule One license was obtained for drug storage and administration. All participants provided written informed consent after receiving a complete description of the study.

### Design

The full study procedure is reported in Carhart-Harris et al. (2016a). The inclusion criteria were major depression of a moderate to severe degree (16+ on the 21-item Hamilton Depression Rating scale [HAM-D]), and no improvement despite two adequate courses of antidepressant treatment. The patients were asked to be antidepressants-free for at least 2 weeks before the study. Twenty patients underwent two psilocybin-assisted therapy sessions, a week apart. The first involved a low-dose of psilocybin (10 mg, p.o.), and the second, a high-dose (25 mg, p.o.). Post capsule ingestion, patients lay with eyes closed and listened to music pre-selected by the research team (Kaelen et al., 2017) (https://www.mixcloud.com/MendelKa/playlists/psilocybin-v13/). Two therapists adopted a non-directive, supportive approach, allowing the patient to experience a mostly uninterrupted introspection. Preparation session occurred 1 week before the 10 mg psilocybin dose and the integration session occurred 1-day and 1-week after the 25 mg psilocybin dose. Out of the initial 20 patients, 19 completed the study (6 females; mean age = 44.7 ± 10.9; 27 to 64). Eight more subjects were added to the study since publication of the initial 12 in Carhart-Harris et al. (2016a)—for a full clinical report of the 20 patients see Carhart-Harris et al. (2017).

### Clinical outcomes

Post-treatment ratings of relevant symptomatology were compared against those collected at baseline (before therapy). The main clinical outcome for this analysis was the self-rated 16-item Quick Inventory of Depressive Symptoms (QIDS-SR16 or just “QIDS-SR” for brevity). Five weeks after the 25 mg psilocybin session was chosen as the primary endpoint. The reason for this was that after 5 weeks, the next point of data collection was 3 months, and at this time-point some of the subjects had gone on to receive new treatments, thus confounding potential inferences. The response rate (≥50% reduction in QIDS-SR scores) at the 5 week time point was 47% (*n* = 9). Secondary clinical outcomes were used to further examine the hypothesis that the mystical-type experience relates to positive clinical outcome. These secondary measures were QIDS-SR at 1-day, 1-week, 3-months, and 6-months; Beck Depression Inventory (BDI, original version) at 1-week, 3-months, and 6-months; Clinician rated Hamilton Depression Rating scale (HAM-D) at 1-week; Dysfunctional Attitudes Scale (DAS; measures trait pessimism) at 1-week and 3-months; Spielberger's Trait Anxiety Inventory (STAI) at 1-day, 1-week, 3-months, and 6-months; Life Orientation Test Revisited (LOT-R; measures optimism) at 1-week and 3-months; and Snaith-Hamilton Pleasure Scale (SHAPS; measures anhedonia) at 1-week and 3-months. Standard criteria for meaningful “response” were calculated for the depression rating scales (≥50% from baseline).

### Measures of acute psilocybin session

The altered state of consciousness questionnaire (ASC) (Dittrich, 1998) was used to measure the acute subjective experience. It was completed retrospectively by the patient as the psilocybin session was coming to an end (i.e., ~5–6 h post ingestion). As stated above, the ASC can be divided into 5 (Dittrich, 1998) (94 items), or 11 dimensions (Studerus et al., 2010) (42 items). The 5 dimensions are: OBN, DED, *visionary restructuralization* (VRS), *auditory alterations* (AUA), and *vigilance reduction* (VIR) (n.b. translation from the German original may explain the slightly peculiar choice of terms e.g., “visionary restructuralization”). As noted above, the OBN items were formulated based on six of the nine categories of “mystical experiences” proposed by Stace (1960) (Bodmer et al., 1994; Studerus et al., 2010) in a similar way to the MEQ (Pahnke and Richards, 1966; Maclean et al., 2012). *Dread of ego-dissolution* is considered to probe negative, aversive experiences in which anxiety is a central aspect. *Visionary restructuralization* measures altered perception and meaning including visual hallucinations and synesthesia. The 11 sub-dimensions are made only from OBN, DED and VRS. The OBN sub-dimensions are *experience of unity, spiritual experience, blissful state, insightfulness*, and *disembodiment*. The DED sub-dimensions are *impaired control or cognition*, and *Anxiety*. The VRS sub-dimensions are *complex imagery, elementary imagery, audio/visual synaesthesia*, and *changed meaning of percepts*.

We hypothesized that OBN and DED would predict clinical outcome up to 5 weeks. To test this hypothesis, we used repeated measure ANOVA. (analysis was done in SPSS v24, GLM with repeated measures). Time was the within-subject factor (independent variable), with QIDS-SR as the within-subject dependent variable in baseline, 1-day, 1-week, 5-weeks. OBN and DED were independent variables (covariates in SPSS). OBN-by-Time and DED-by-Time interactions were the primary outcomes of interest. The contrast for the within-subject factor was simple, comparing each level to the 1st one (baseline). Furthermore, we hypothesized specificity in the relationship between OBN and depression changes by comparing the strength of this correlation with that between the perceptual factors from the ASC, namely VRS and AUA, and depression changes (Steiger, 1980; Lee and Preacher, 2013). A threshold of OBN > 0.6 was used to distinguish a “complete” OBN. This threshold is similar to MEQ > 0.6 which was used in other studies to identify “peakers” and “complete mystical-type experience” (Pahnke et al., 1970; Richards et al., 1977; Maclean et al., 2011; Garcia-Romeu et al., 2014; Johnson et al., 2016). In a different study, OBN and MEQ showed a Pearson correlation of 0.93 (Liechti et al., 2017), suggesting that these two questionnaire quantify a similar experience and that a similar threshold can be used. For descriptive purposes, we tested whether those patients who had a “complete” OBN had a better clinical outcome. This analysis was done to expand the initial hypothesis to other time points and questionnaires.

We also issued participants an in-house measure, the 29-item “psychedelic questionnaire” (PQ)—which was completed at the same time as the ASC. The PQ has been previously used in a number of our pharmacological challenge studies due to its brevity relative to the full ASC (Carhart-Harris et al., 2012, 2016b). As a descriptive exploratory analysis, correlation between PQ and clinical outcome at 5 weeks was calculated for all items. The same exploratory analysis was also done on all of the 94 items of the ASC.

## Results

### Prediction of QIDS-SR up to 5 weeks

**Figure 1:**
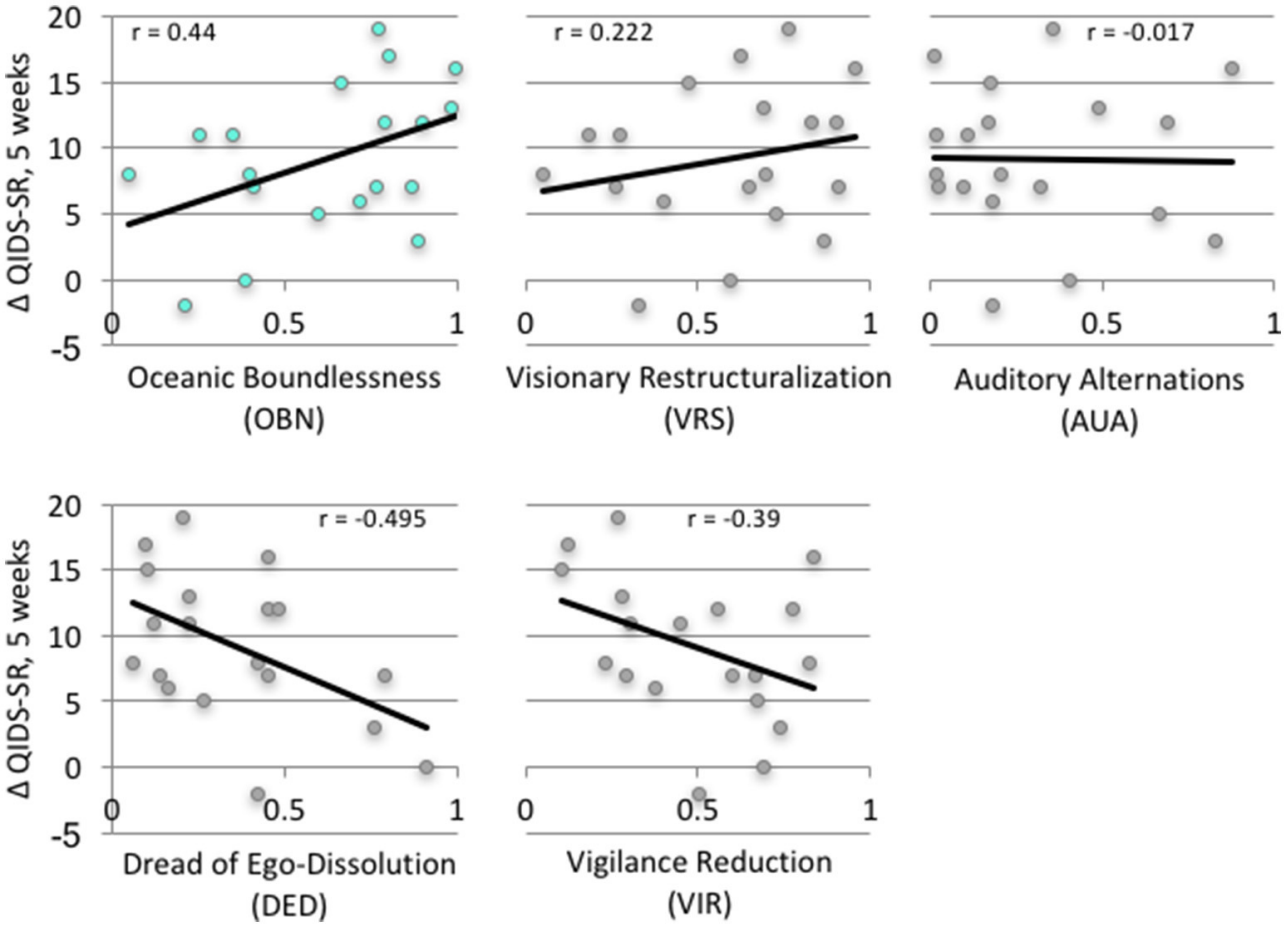
Correlation of ASC (5 dimensions) with change of clinical outcome at 5 weeks (ΔQIDS-SR).

**Figure 2:**
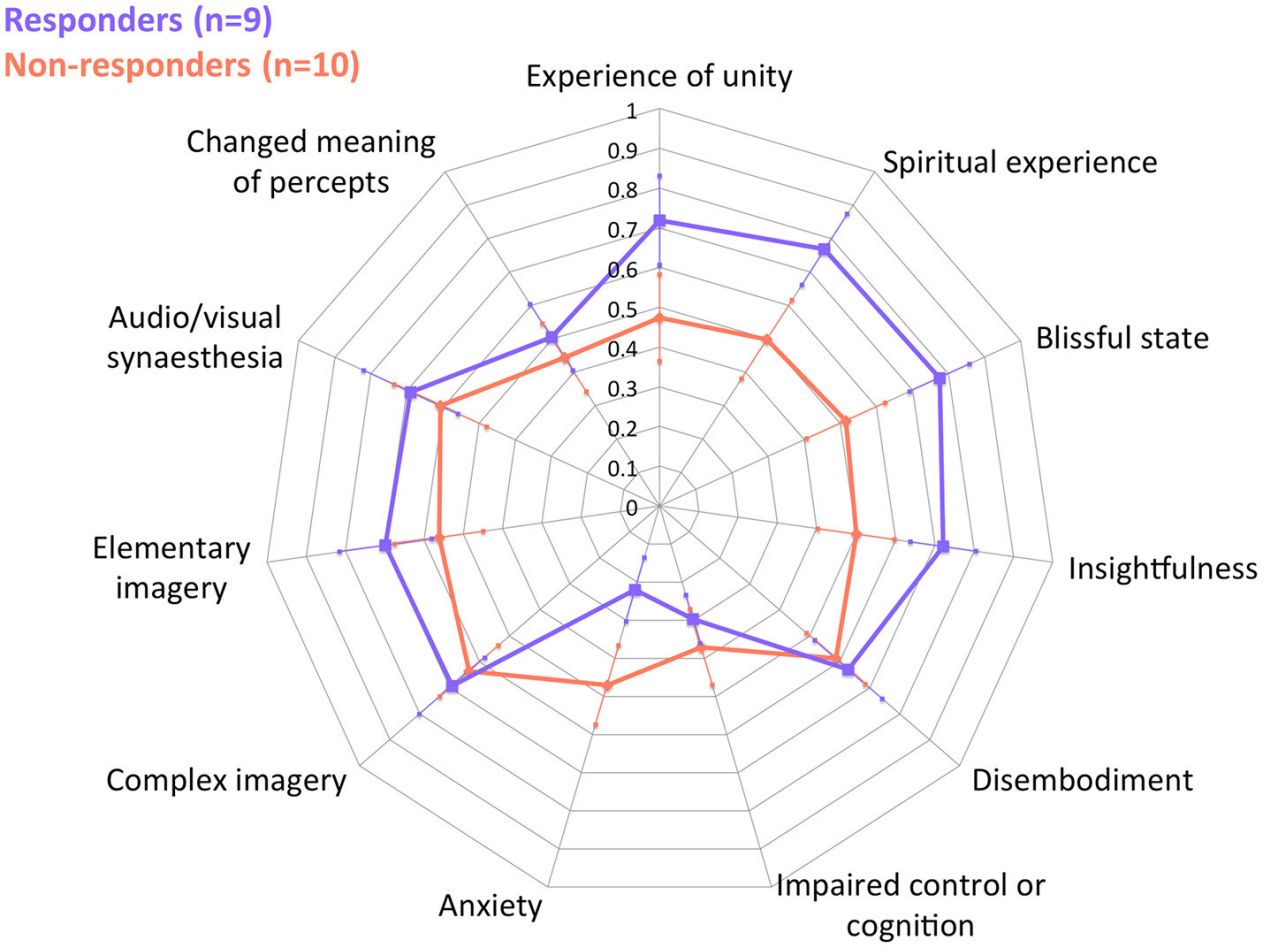
ASC (11 dimensions) of responders and non-responders at 5 weeks. Error Bars = Standard Error.

These following are primary results of this study. Table 1 presents the results of the repeated measures ANOVA with Time as the within-subject factor (independent variable), QIDS-SR as the within-subject dependent variable in baseline, 1-day, 1-week, 5-weeks. OBN and DED were independent variables. [Sphericity assumed: Mauchly's W = 0.71 (*p* = 0.411)]. For the interactions of Time X OBN, and Time X DED, the within-subjects effect and the within-subjects contrasts at each time point compared to baseline were all significant (*p* < 0.05), confirming our main hypothesis. Regression analysis with ΔQIDS-SR (5-weeks) as a dependent variable and OBN and DED as independent variables found that together they explain 54% of the variance (*r*^2^ = 0.59, adjusted *r*^2^ = 0.54; standardized beta values of OBN, DED, were 0.605, −0.649, respectively). For descriptive purposes, Figure 1 presents plots of Pearson's correlation of the 5 dimensions of the ASC predicting ΔQIDS-SR (5 weeks). Furthermore, based on a standard threshold for defining clinical response (≥50% reduction in QIDS-SR score at 5 weeks vs. baseline), a comparison of responders (*n* = 9) vs. non-responders (*n* = 10) in the 11D ASC scores is presented for descriptive purposes in Figure 2.

**Table 1:** Repeated measures ANOVA; OBN and DED predict changes in QIDS-SR over different time points up to 5 weeks.

**Within-subjects effects**		***F*_(3, 48)_**	***p***	**Partial η^2^**
Time ^*^ OBN	Sphericity Assumed	5.563	0.002	0.258
Time ^*^ DED	Sphericity Assumed	15.39	0.003	0.252
**Within-subjects contrasts**		***F***_(1, 16)_	***p***	**Partial** η^2^
Time ^*^ OBN	1 Day vs. Baseline	13.143	0.002	0.451
	1 Week vs. Baseline	7.237	0.016	0.311
	5 Weeks vs. Baseline	13.29	0.002	0.454
Time ^*^ DED	1 Day vs. Baseline	9.941	0.006	0.383
	1 Week vs. Baseline	6.828	0.019	0.299
	5 Weeks vs. Baseline	15.298	0.001	0.489

*Time is the within-subject factor (independent variable), QIDS-SR is the within-subject dependent variable in baseline, 1-day, 1-week, 5-weeks. OBN and DED are independent variables. Time-by-OBN and Time-by-DED interactions are the primary outcomes of interest (within-subjects effects). Within-subjects simple contrasts are calculated for each time point compared to baseline. A significant within-subject contrast suggests a linear relationship between OBN or DED and the difference between time points in QIDS-SR. Sphericity assumed: Mauchly's W = 0.71 (p = 0.411). partial η^2^ > 0.25 = large effect size. To inform directionality see Figure 1 for Pearson's correlations: high OBN and low DED predict reductions in QIDS-SR*.

As hypothesized, OBN was a significantly better predictor of reductions in depression than both *VRS* and *AUA* (*z* = 1.64 and *z* = 2.01, respectively, *p* < 0.05) (Steiger, 1980; Lee and Preacher, 2013).

### Prediction of secondary clinical measures

The following are the secondary results of this study. Clinical outcomes for “complete” OBN were compared with those for “non-complete” OBN for secondary clinical outcomes such as measures of trait anxiety, anhedonia, optimism and pessimism (Table 2). Patients that had “complete” OBN (*n* = 11, OBN = 0.83 ± 0.1) had better outcomes than those who did not (*n* = 8, OBN = 0.33 ± 0.16), on a number of different measures and at different time points (1-day, 1-week, 5-weeks, 3-months, and 6-months). Response rates of “complete” OBN are presented in Table 2.

**Table 2:** Comparisons of “complete” OBN (*n* = 11) and “non-complete” (*n* = 8) with different clinical measures in different time points.

		**Complete vs. Non**	**Mean** Δ		**Response rate**	
		**Cohen's *d***	**Complete (*n* = 11)**	**Non (*n* = 8)**	**Complete (*n* = 11)**	**Non (*n* = 8)**
1 Day	ΔQIOS-SR	1.39	13.18 ± 4.4	8.25 ± 5.6	81.8	50
	ΔSTAI	0.94	25.18 ± 12.9	15 ± 17.4		
1 Week	ΔQIOS-SR	1.18	12 ± 4.6	7.75 ± 5.6	81.8	37.5
	ΔBDI	0.61	24.45 ± 7.3	19.25 ± 15.5	81.8	37.5
	ΔHAM-D	1.40	17.81 ± 5	10.62 ± 8.9	90.9	25
	ΔSTAI	0.85	27.72 ± 12.3	18.5 ± 18		
	ΔDAS	2.10	44.54 ± 22.9	12.87 ± 19.7		
	ΔSHAPS	−0.07	4.54 ± 3.7	4.75 ± 4.9		
	ΔLOT-R	1.40	7.63 ± 5.2	1.75 ± 6.6		
5 Weeks	ΔQIOS-SR	1.58	11.54 ± 5.1	6 ± 4.8	63.6	25
3 Months	ΔQIOS-SR	1.17	9.45 ± 5.5	4.12 ± 7.3	54.5	12.5
	ΔBDI	1.38	20.45 ± 12	8.12 ± 13.3	72.7	12.5
	ΔSTAI	0.79	15.18 ± 11.1	8 ± 14.3		
	ΔDAS	2.22	39.72 ± 24	−1.25 ± 28.1		
	ΔSHAPS	0.81	4.45 ± 3	1.75 ± 6		
	ΔLOT-R	1.13	4.36 ± 3.9	0.75 ± 5		
6 Months	ΔQIOS-SR	0.96	9.18 ± 6.4	4.37 ± 7.7	45.5	25
	ΔBDI	1.25	19.54 ± 9.7	8.62 ± 14.6	63.6	25
	ΔSTAI	0.15	16.36 ± 12.5	14.87 ± 16.1		

*The difference between “complete” OBN and non-complete in effect size (cohen's d > 0.8 = large effect); the mean change of each group; and the response rates (%) of each group for depression questionnaires only. BDI, Beck Depression Inventory; DAS, Dysfunctional Attitudes Scale (measures trait pessimism); HAM-D, Clinician rated Hamilton Depression Rating scale; LOT-R, Life Orientation Test Revisited (measures optimism); QIDS-SR, Self-Reported Quick Inventory of Depressive Symptoms; SHAPS, Snaith-Hamilton Pleasure Scale; STAI, Spielberger's Trait Anxiety Inventory*.

In further exploratory analyses, correlations were calculated between all 94 items of the ASC and ΔQIDS-SR and were ordered by the strength of correlation (Table S1). The same was done for all 29 items of the PQ (Table S2). In both examples, it is apparent that items that best relate to OBN correlate most strongly with positive clinical outcomes, while sensory phenomena correlate less, and anxiety is predictive of worse outcomes.

## Discussion

Consistent with our prior hypothesis, psilocybin-induced high OBN (sharing features with mystical-type experience) and low DED (similar to anxiety) predicted positive long-term clinical outcomes in a clinical trial of psilocybin for TRD. This result replicates those of previous studies showing that psychedelic-induced peak or mystical-type experiences are predictive of positive long-term outcomes (O'Reilly and Funk, 1964; Klavetter and Mogar, 1967; Pahnke et al., 1970; Kurland et al., 1972; Richards et al., 1977; Maclean et al., 2011; Bogenschutz et al., 2015; Griffiths et al., 2016; Johnson et al., 2016; Ross et al., 2016). This relationship appears to be somewhat specific, in that OBN was significantly more predictive of positive clinical outcomes than altered visual and auditory perception—endorsing the moniker “psychedelic” (“mind-revealing”) over “hallucinogen” when referring to this class of drug—at least in the context of psychedelic therapy. It also suggests that the therapeutic effects of psilocybin are not a simple product of isolated pharmacological action but rather are *experience dependent*. We also found that greater DED (anxiety and impaired cognition) experienced during the drug session was predictive of less positive clinical outcomes.

One may naturally infer from these findings that the occurrence of OBN or mystical-type experience mediates long-term positive clinical outcomes (Griffiths et al., 2016; Ross et al., 2016) and while this assumption may be valid, we must exercise caution about ascribing too much to this relationship. It remains possible that as yet unmeasured and therefore unaccounted for components of psychedelic therapy play important roles in mediating long-term outcomes. There are several candidate factors in this regard, and the following should not be considered an exhaustive list: *emotional insight*/*breakthrough* or *catharsis*; *priming* and *suggestibility*; *reliving of trauma*/*defining life events*; *insights* about the self and relationships; the patients relationship to *music* heard; his/her success at “*letting go*”; the quality of *therapeutic relationship*; and the degree of “*closure*” attained during post-drug *integration work* (Frederking, 1955; Sandison, 1955; Abramson, 1956; Martin, 1957; Eisner and Cohen, 1958; Leuner, 1961; Jensen, 1963; Shagass and Bittle, 1967; Richards, 1978; Loizaga-Velder, 2013; Gasser et al., 2014; Belser et al., 2017; Russ and Elliott, 2017; Watts et al., 2017).

These factors may exert influence before, during and after the acute experience itself and may also be more or less dependent on particular psychological frameworks and their relevant vocabularies. For example, the psychoanalytic models of Freud and Jung were dominant in psychiatry in the mid-twentieth century and thus references to *ego, repression* and *the unconscious* are commonplace among the psychedelic research literature of this period. While the processes that underlie these constructs may indeed be operative in the context of psychedelics, little effort has been made to define, measure and quantify their contributions (Shagass and Bittle, 1967; Barr et al., 1972). The development of subjective (Nour et al., 2016), behavioral and biological measures (Carhart-Harris et al., 2012, 2016b; Lebedev et al., 2015; Tagliazucchi et al., 2016) relevant to these constructs, and more importantly, the processes that underlie them, would represent an important advance not just for psychedelic science but for the psychological frameworks themselves (Carhart-Harris et al., 2014). We should be conscious of not being too attached (or averse) to any specific theoretical frameworks however, and approaches that endeavor to access “framework-free” descriptions of phenomena may prove particularly useful in this regard (Varela, 1996; Petitmengin, 2006). Critically, it is our view that it is possible to work toward a secular, biologically-informed account of the mystical-type experience that does not resort to “explaining away” or “reducing down” the core phenomenology and depth psychology may be a useful bedfellow in this regard.

Returning to the present study's main findings, DED was found to negatively correlate with clinical outcome, yet, none of the patients showed a worsening of clinical symptoms at 5 weeks. Less DED combined with high OBN predicted 54% of the variance of clinical change at 5 weeks—a substantial contribution and one that helps justify the emphasis placed on minimizing anxiety and relinquishing psychological resistance in psychedelic therapy (Eisner and Cohen, 1958; Sherwood et al., 1962; Grof et al., 2008; Richards, 2015), as well as paying careful attention to preparation and “set and setting” (Hartogsohn, 2017; Carhart-Harris et al., in press). That anxiety arises in parallel with psychological struggle is resonant with principles of psychoanalytic theory (Sandison, 1961), as can be seen in the choice of terms for the “DED” and “OBN” factors of the ASC—both of which invoke constructs that can be traced to Freud (1929, 1962).

According to psychoanalytic theory, the overcoming of psychological resistance is required for emotional breakthrough and insight (Freud, 1920) and the occurrence of mystical-type/peak experiences (Jung, 2014). Consistently, writers on the mystical-type/peak experience have reliably identified loss of self or “ego-dissolution” as one of its basic pre-requisites and features (James, 1902; Stace, 1960; Maslow, 1964). Recent work has sought to develop and validate a measure that is sensitive to difficult or challenging psychedelic experiences (Barrett et al., 2016; Carbonaro et al., 2016) and there is some evidence that the intensity of such experiences is predictive of positive long-term outcomes, whereas the duration of struggle is predictive of negative outcomes (Carbonaro et al., 2016). This is presumably because the successful resolution of conflict brings with it, insight and relief, whereas the failure to breakthrough perpetuates suffering. ASC and other questionnaires such as the challenging experience questionnaire (CEQ) (Barrett et al., 2016) may be insensitive to whether or not successful resolution of psychological conflict has occurred. Therefore, the development of new scales specifically designed to focus on *emotional breakthrough* after struggle may add considerable value.

Improving our subjective measures of high-level human experiences such as the mystical-type/peak experience will enhance our ability to understand their psychology and underlying neural substrates. As touched on in the introduction, psychopharmacology is increasingly acknowledging the importance of “context” and particularly “environment” as a factor mediating the effects of both intrinsic neurobiological features (e.g., genotypes) and exogenous pharmacological inputs—such as drugs (Alexander et al., 1981; Caspi et al., 2010). For example, a recent popular model of the action of SSRIs incorporates “environment” and cognitive (re)appraisal (Harmer et al., 2017) as key determinants of therapeutic efficacy (see also Branchi, 2011; Belsky, 2016). Like SSRIs, classic psychedelic drugs also work on the serotonin system; however, unlike the SSRIs, they are direct agonists at the 5-HT2A receptor (Nichols, 2016). There is compelling evidence that the 5-HT2A receptor is psychedelics' key site of action (Nichols, 2016). Intriguingly, recent work has found that the phenotypic expression of 5-HT2AR genotypes is significantly dependent on the influence of “environment” (Jokela et al., 2007). These findings may imply that enhanced sensitivity to context is an important function of 5-HT2A receptor signaling (Carhart-Harris and Nutt, 2017).

Ascending from the pharmacological to the whole-brain systems level, increased cortical entropy has been found to be a reliable feature of the psychedelic state (Carhart-Harris et al., 2014), to relate to high-level subjective experiences such as “ego-dissolution” (Nour et al., 2016; Atasoy et al., 2017; Schartner et al., 2017) that are relevant to the mystical-type experience, and to be predictive of longer-term trait changes—such as increased “openness” (Lebedev et al., 2016). Recent work suggests that increased brain entropy under psychedelics is consistent with the brain being more closely tuned to “criticality” (Atasoy et al., 2017). Criticality refers to systems that reside in a functional “sweet spot”, critically poised between order and disorder—in which they can effectively retain information (by being sufficiently ordered) while being appropriately adaptive and sensitive to change (by being sufficiently disordered). Intriguingly, one of the signatures of a critical system is a sensitivity to perturbation (Bak, 1996). It follows that enhanced sensitivity to perturbation in a psychedelically-induced “entropic” and “critical” brain may account for the special sensitivity to “environment” that is characteristic of the psychedelic state (Hartogsohn, 2016; Carhart-Harris et al., in press).

Understanding the neurobiological mechanisms of OBN, mystical-type or peak experiences (Vollenweider, 2001) should enable us to better comprehend, define and study them. This is important, not least because they are proving to be important determinants of treatment success in psychedelic therapy (Richards et al., 1977; Bogenschutz et al., 2015; Griffiths et al., 2016; Johnson et al., 2016; Ross et al., 2016). Crucially, better understanding the biological basis of mystical-type/peak experiences and their longer-term impact on the mind and brain should help to demystify them, facilitating an easier conversation about them with mainstream psychology. Researchers in the mainstream have as much a responsibility as those in “the periphery” to facilitate this. Denying the relevance of these phenomena is as damaging to scientific progress as denying their physical basis. The prize for successfully integrating mystical-type experience into mainstream science may be their potential to have a substantial positive impact on medicine, education and society—which ironically, may, at least in part, explain why their integration into western society has proved so difficult to achieve (Stevens, 1987).

To summarize, the occurrence of high OBN (sharing features with mystical-type experience) and low DED (relating to anxiety and impaired cognition) under psilocybin predicted positive clinical outcomes in a trial of psilocybin for TRD. This relationship exhibited a degree of specificity, in that psilocybin-induced OBN was significantly more predictive of reduced depressive symptoms than the drug's more generic visual and auditory perceptual effects. Future work, with a larger sample size, is required to more comprehensively and systematically measure the influence of different potential predictive factors on the quality of acute psychedelic experiences (Gasser et al., 2014; Belser et al., 2017; Watts et al., 2017) and subsequent long-term outcomes (Carhart-Harris et al., in press). As psychedelic therapy gains influence and credibility (Carhart-Harris and Goodwin, 2017), it seems vital that appropriate consideration is paid to the importance of promoting a certain kind of experience, as the quality of that experience may be *the* critical determinant of therapeutic success.

## Author contributions

LR analyzed the data and wrote the paper; DN sanctioned the research and approved an earlier draft of the manuscript; RC-H designed and conducted the research, and wrote the paper.

### Conflict of interest statement

The authors declare that the research was conducted in the absence of any commercial or financial relationships that could be construed as a potential conflict of interest.
