# Supplementary material for: Quality of Acute Psychedelic Experience Predicts Therapeutic Efficacy of Psilocybin for Treatment-Resistant Depression
Source: Front Pharmacol. 2018 Jan 17;8:974. doi: 10.3389/fphar.2017.00974 (PMC5776504; doi:10.3389/fphar.2017.00974)
Supplement: Supplementary file 1 [file Table1.pdf]

Supplementary Table S1

Quality of acute psychedelic experience predicts therapeutic efficacy of psilocybin for treatment-resistant depression

Leor Roseman[1], David J Nutt[1] & Robin L Carhart-Harris[1]

*** Correspondence**
Leor Roseman
leor.roseman13@imperial.ac.uk

Table S1. Correlations between ASC items and clinical outcome in 5 weeks compared to baseline (ΔQIDS-SR), ordered by strength. Due to the exploratory nature of this table, p value are not corrected for multiple comparisons. The colors of the items are based on their 5D-ASC factors: Blue = Oceanic Boundlessness (OBN); Red = Dread of Ego Dissolution (DED); Yellow = Visionary Restructuralization (VRS); Green = Auditory Alternations (AUA); Grey = Vigilance Reduction (VIR).

ASC	Pearson's r	p (2-tail)
I felt particularly profound.	0.581	0.005
I had particularly inventive ideas.	0.553	0.007
I experienced a profound inner peace	0.541	0.008
Worries and fears of everyday life felt irrelevant.	0.526	0.010
I felt one with my surroundings.	0.523	0.011
I experienced past, present, and future as a unity.	0.520	0.011
Oppositions and contradictions seemed to resolve.	0.499	0.015
Many things appeared incredibly funny to me.	0.477	0.019
I felt connected to a higher power.	0.469	0.021
I experienced boundless joy.	0.460	0.024
I thought I would lastingly change in a wonderful way.	0.445	0.028
A voice commented on everything I thought although no one was there.	0.443	0.029
I felt full of awe.	0.409	0.041
Body sensations were full of enjoyment.	0.397	0.046
I had insights into the ways the world works that were mysterious to me before.	0.389	0.050
My experience had a religious character.	0.374	0.057
Many things appeared to me as breathtakingly beautiful.	0.372	0.058
All things seemed to unify as a whole.	0.367	0.061

I felt like I was floating.	0.360	0.065
The boundaries between me and my surroundings seemed to vanish.	0.350	0.071
I felt completely free and detached from all obligations.	0.347	0.073
I saw colors in complete darkness or with closed eyes.	0.341	0.076
I experienced all-embracing love.	0.329	0.084
The world seemed to me beyond good and evil.	0.312	0.097
I experienced a glimpse of eternity.	0.271	0.131
I felt like I was in a wonderful other world.	0.260	0.141
My sense of time and space was altered as if I was dreaming.	0.258	0.143
I could see images from my memory or imagination with exceeding clarity.	0.244	0.157
Everything around me seemed to be animated with life.	0.243	0.158
Meaningless noises sounded like real words or phrases to me.	0.229	0.173
I saw brightness or flashes in complete darkness or with closed eyes.	0.224	0.178
I heard my thoughts as if I had spoken out loud.	0.221	0.182
Colors seemed to be altered by sounds or noises.	0.191	0.216
My thoughts and actions were slowed down.	0.185	0.224
Shapes seemed to be changed by sounds or noises.	0.180	0.230
Things came to my mind that I thought long forgotten.	0.173	0.239
Sounds seemed to influence what I saw.	0.171	0.242
I saw regular patterns in complete darkness or with closed eyes.	0.171	0.242
I saw whole scenes in complete darkness or with closed eyes.	0.144	0.278
I heard full sentences without knowing where they came from.	0.133	0.294
I felt like a doll on a string or a marionette.	0.105	0.334
Everything happened so fast that I could not follow it all.	0.099	0.343
My imagination was extremely vivid.	0.099	0.344
I felt extraordinary forces within me.	0.096	0.348
I saw things I knew were not real.	0.094	0.351
Things in my surroundings had a new or alien meaning.	0.039	0.437
Things in my surroundings appeared smaller or larger.	-0.015	0.476
I heard rings and tones without knowing where they came from.	-0.015	0.475
I heard music without knowing where it came from.	-0.018	0.470
I heard buzzing, swooshing, or humming without recognizing the cause.	-0.034	0.444
Some unimportant things acquired special meaning.	-0.052	0.417
I felt isolated from everything and everyone.	-0.062	0.400

I could remember certain events with exceeding clarity.	-0.064	0.398
I heard single words without knowing where they came from.	-0.072	0.384
Objects in my surroundings touched me more emotionally.	-0.089	0.358
I heard something faintly that I could not identify.	-0.092	0.354
I heard voices that did not come from the surroundings as usual.	-0.099	0.344
There were sounds in the room that I feel were unlikely to have been real.	-0.135	0.290
Sounds and noises were less intense than normally.	-0.135	0.290
I had the impression I was out of my body.	-0.138	0.287
From an initially diffuse noise, which I could not identify as real, clear rings and tones evolved.	-0.152	0.268
I heard diffuse noises without knowing where they came from.	-0.155	0.264
I felt like I do shortly before falling asleep.	-0.156	0.262
I could not get a melody out of my mind.	-0.157	0.261
I had difficulties in distinguishing important from non-important.	-0.185	0.224
I felt sleepy.	-0.191	0.217
My body seemed numb, lifeless, or alien.	-0.207	0.197
I felt disembodied.	-0.222	0.181
I heard a ticking, knocking, ringing, or rattling without being able to recognize the cause.	-0.228	0.174
I felt exhausted.	-0.260	0.141
My surroundings appeared strange and eerie.	-0.267	0.135
My perception was hazy	-0.274	0.128
I felt that I was on the verge of fainting.	-0.275	0.127
I was incapable of making even the slightest decision.	-0.277	0.125
I perceived everything blurry, as if through a kind of fog.	-0.296	0.110
I had the impression of no longer having my own will.	-0.316	0.094
I felt dozy.	-0.323	0.089
I thought I was about to fall asleep.	-0.328	0.085
I stayed in a very unnatural pose for an extended period of time.	-0.343	0.075
I was afraid of losing control over myself.	-0.395	0.047
I felt dazed.	-0.401	0.045
I was afraid of not being able to get out of the state in which I found myself.	-0.444	0.028
I felt drunk.	-0.467	0.022
I felt as if dark forces had overtaken me.	-0.488	0.017
My thoughts were always interrupted; I could not think anything to its end.	-0.511	0.013

I was scared without knowing why.	-0.519	0.011
I felt paralyzed.	-0.529	0.010
I experienced everything as frighteningly distorted.	-0.551	0.007
I felt as if I was half-asleep.	-0.562	0.006
I felt tortured.	-0.576	0.005
I had the feeling that something terrible was going to happen.	-0.586	0.004
I had the feeling of unbearable emptiness.	-0.587	0.004
I felt threatened.	-0.607	0.003
Time passed slowly in a painful way.	-0.616	0.002
