# Supplementary material for: Quality of Acute Psychedelic Experience Predicts Therapeutic Efficacy of Psilocybin for Treatment-Resistant Depression
Source: Front Pharmacol. 2018 Jan 17;8:974. doi: 10.3389/fphar.2017.00974 (PMC5776504; doi:10.3389/fphar.2017.00974)
Supplement: Supplementary file 2 [file Table2.pdf]

Supplementary Table S2

Quality of acute psychedelic experience predicts therapeutic efficacy of psilocybin for treatment-resistant depression

Leor Roseman[1], David J Nutt[1] & Robin L Carhart-Harris[1]

*** Correspondence**
Leor Roseman
leor.roseman13@imperial.ac.uk

Table S2. Correlation between PQ items and change of clinical outcome at 5 weeks (ΔQIDS), ordered by strength. The table is provided for descriptive purposes, p values are not corrected for multiple comparisons.

PQ item	Pearson's r	p (2-tail)
I felt an inner warmth	0.616	0.002
I felt amazing	0.591	0.004
I felt a profound inner peace	0.540	0.008
I felt loved up	0.517	0.012
I felt really sharp	0.507	0.013
I felt energised and enthusiastic about things	0.467	0.022
My thoughts wandered freely	0.372	0.059
I felt like I was floating	0.322	0.090
The experience had a spiritual or mystical quality	0.259	0.142
Sounds influenced things I saw	0.166	0.249
I experienced a disintegration of my 'self' or 'ego'	0.155	0.264
I saw geometric patterns	0.146	0.276
I experienced a sense of merging with my surroundings	0.110	0.327
My imagintaion was extremely vivid	0.074	0.382
Edges appeared warped	0.025	0.459
The experience had a supernatural quality	-0.020	0.468
My perception of time was distorted	-0.030	0.451
I saw scenes from my past	-0.060	0.404
How intense were the drug effects when at their peak ?	-0.105	0.334
My sense of size and space was distorted	-0.161	0.255
I saw movement in things that weren't really moving	-0.161	0.255
Things looked strange	-0.163	0.253
The experience had a dream like quality	-0.172	0.241
I felt unusual bodily sensations	-0.276	0.127
My thinking was muddled	-0.451	0.026
I felt entirely normal	-0.460	0.024
I feared losing control of my mind	-0.513	0.012
I felt suspicious and paranoid	-0.540	0.008
I felt afraid	-0.651	0.001
